# An integrative review of associations between polymorphic variants and the metabolic syndrome

**DOI:** 10.1590/1677-5449.007917

**Published:** 2018

**Authors:** Jamille Silva Oliveira, Rita Narriman Silva de Oliveira Boery

**Affiliations:** 1 Universidade Estadual do Sudoeste da Bahia – UESB, Programa de Pós-graduação em Enfermagem e Saúde – PPGES, Jequié, BA, Brasil.

**Keywords:** genetic polymorphism, metabolic syndrome, hypertension, obesity, insulin resistance, dyslipidemias

## Abstract

The pathogenesis of metabolic syndrome, i.e. of each of its components, is complex and has not been entirely elucidated. As a result, it is very difficult to establish a definition of which clinical factors are the most important determinants of its development. The objective of this review is to describe Brazilian scientific research investigating associations between the metabolic syndrome and genetic factors. We selected fifteen studies that met the inclusion and exclusion criteria. Our analysis revealed that there is a modest volume of Brazilian studies investigating relationships between genes, their polymorphic variants and the metabolic syndrome and its risk factors. Therefore, more studies are needed to better understand the biological roles played by genetic polymorphisms and their relationships with metabolic syndrome or its risk factors.

## INTRODUCTION

 Metabolic syndrome (MS) is used as a clinical tool for identifying patients with metabolic risk of cardiovascular diseases. 1 In general, MS can be classified as a group of metabolically interrelated pathophysiologic cardiovascular risk factors that have origins in complex disorders and are subject to both environmental and genetic influences. 2 ^,^ 3 The pathogenesis of MS, i.e., of each of its components, is complex and has not been entirely elucidated. As a result, it is very difficult to establish a definition of which clinical factors are the most important determinants of its development. Nevertheless, there is consensus on the principal components of MS that are associated with increased cardiovascular morbidity and mortality: excess weight, elevated arterial blood pressure, and disorders of sugar and lipid metabolism. 2 ^,^ 4 ^-^ 7

 Since it is a multifactorial disorder, both the genetic factor and the environmental factor are important to understanding the interactions between all of the components of risk of development of MS. Moreover, many different gene loci are involved in expression of the components of energy metabolism, which makes it a very complex task to determine the influence of gene-environment and gene-gene interactions on each of the different cardiovascular risk factors. 8

 The most common class of markers in the human genome are small variations in DNA sequences caused by localized substitution mutations, known as single nucleotide polymorphisms (SNPs) and accounting for around 90% of sequence differences. 9 There will be 3 million differences between any two people in their roughly 10,0000 different amino acids. 10 However, the great majority of single nucleotide mutations are rare in the population, and a polymorphism must have a frequency of at least 1% of the population to be considered an SNP. 

 One of the principal objectives of research into SNP is to understand the genetics of human phenotypical variations and particularly the genetic bases of complex human diseases. 11 ^,^ 12 Genetic factors can explain part of the increase in cardiovascular risk, 13 because many different genes are involved in controlling the different metabolic pathways. The mechanisms controlled by gene alleles include inflammatory processes, platelet activity, neurohormonal activity (renin-angiotensin system), lipid metabolism, and oxidative stress. 14

 Genome studies have led to identification of many genetic markers of cardiovascular risk/protection in different populations around the world, notably the genes IL1RA, CD14, PON1, ALOX5AP, loci 9p 21.3, and *PON1* (Met55Leu and Gln192Arg). 15 ^-^ 18 Furthermore, studies suggest that the roles played by these cardiovascular risk factors may vary between ethnic groups. 19 ^-^ 22 These studies indicate that genetic polymorphisms distributed differently in populations of different ethnicities play a fundamental role in the presence of the ethnic/racial differences observed in MS. 

 Against this background, the objective of this study is to profile Brazilian scientific output describing studies of the associations between MS and its genetic factors. 

## METHODOLOGY

 This is an integrative review of the literature, of a descriptive and exploratory character, based on the results of electronic searches for academic publications on the subject of current genetic research into MS in Brazil. 

 The choice of databases to be searched took into consideration the fact that each has its own peculiarities, area of concentration, and focus. The databases chosen were those considered relevant to the subject investigated: SciELO® (Scientific Electronic Library Online*)* , PubMed Central® (developed and maintained by the National Center for Biotechnology Information, NCBI), and the Biblioteca Virtual em Saude (BVS) Brazil. In addition to these databases, Google Scholar® was also consulted to give a wider overview of publications and provide a basis for comparison with the results from the other databases. 

 The electronic searches were conducted during June of 2017 and the search strategies were defined after selection of the databases. The keywords “polymorphism”, “metabolic syndrome”, and “Brazil” were used, combined using Boolean logical operators to refine and specify the searches. The keywords were used in Portuguese and English, depending on the requirements of each database. 

 Studies were selected according to the following inclusion criteria: 1) publications in magazines or journals; 2) publications that discussed the main subject (polymorphisms and MS); and 3) work published during the preceding 5 years (2013 to 2017). The criterion for exclusion was title, keywords, and abstract that did not cover the subject and did not meet the inclusion criteria. 

 After selection of studies, according to the inclusion and exclusion criteria, information from them was input to a form designed for recording the data collected. This instrument was created in a spreadsheet to facilitate organization, grouping, and analysis of the data, interpretation of the results, and presentation of the review. It comprises columns for insertion of reference, category, objective, methodology, results, and conclusions. 

## RESULTS

 Fifteen studies were selected for the review. All of them were conducted with the general objective of investigating relationships between genes and their polymorphic variations and MS and its risk factors (obesity, insulin resistance, hypertension, and lipid profile abnormalities). The genes investigated to test for associations were NOS, MMP-2, IL6R, VDR, UCP1, ADRB3, ApoE, IRS-1, PPARG, APOA5, LEP, LEPR, NR3C1, GR, IL1B, IL2, IL4, IL8, IL10, IFNγ, TNFa and ACE. 

 The first search was run on all four databases using just the keywords with Boolean operators: “polymorphism” AND “metabolic syndrome” AND “Brazil”. The database that returned the fewest results was SciELO, with just one article, followed by BVS with 17, PubMed with 38, and Google Scholar with 4,430. In order to adapt and refine the analysis, searches were run again with a filter to restrict results to the preceding 5 years (2013-2017). It will be observed that Google Scholar® returned the highest number of studies, which is because its search metric is different to those used by the other databases. This database was therefore used to identify possible differences between the results generated by the others, since each has its own metric, which can lead to different results, as can articles contained in one database but not in another. 

 After filtering by availability and reading, consisting of reading the title, abstract, and keywords of each study, 15 articles were selected ( Table 1 ). 23 ^-^ 37 the remainder were excluded from the analysis, because they were not aligned with the subject of the review or with the inclusion parameters. 

**Table 1:** Studies selected after application of selection criteria.

**Authors/data**	**Title**	**Journal**	**Database**
Vargas et al. (2013) 23	Influence of the 48867A>C (Asp358Ala) IL6R polymorphism on response to a lifestyle modification intervention in individuals with metabolic syndrome	Genet Mol Res	PubMed & BVS
Belo et al. (2013) 24	Effect of metabolic syndrome risk factors and MMP-2 genetic variations on circulating MMP-2 levels in childhood obesity	Mol Biol Rep	PubMed
Brondani et al. (2014) 25	The presence of at least three alleles of the ADRB3 Trp64Arg (C/T) and UCP1-3826A/G polymorphisms is associated with protection to overweight/obesity and with higher high-density lipoprotein cholesterol levels in Caucasian-Brazilian patients with type 2 diabetes	Metab Syndr Relat Disord	PubMed
Teixeira et al. (2015) 26	Association of IL-6 polymorphism -174G/C and metabolic syndrome in hypertensive patients	Biomed Res Int	PubMed
Almeida et al. (2017) 27	Different metabolic responses induced by long-term interdisciplinary therapy in obese adolescents related to ACE I/D polymorphism	J Renin Angiotensin Aldosterone Syst	PubMed
Martins et al. (2017) 28	HPA axis dysregulation, NR3C1 polymorphisms and glucocorticoid receptor isoforms imbalance in metabolic syndrome	Diabetes Metab Res Rev	PubMed
Schuch et al. (2013) 29	Relationship between Vitamin D Receptor gene polymorphisms and the components of metabolic syndrome	Nutr J	PubMed & BVS
Gelaleti et al. (2015) 30	IRS-1 gene polymorphism and DNA damage in pregnant women with diabetes or mild gestational hyperglycemia	Diabetol Metab Syndr	PubMed
Franca et al. (2016) 31	APOA5 polymorphisms associated with lipid metabolism in Brazilian children and adolescents	Genet Mol Res	PubMed
Maintinguer Norde et al. (2017) 32	Influence of IL1B, IL6 and IL10 gene variants and plasma fatty acid interaction on metabolic syndrome risk in a cross-sectional population-based study	Clin Nutr	PubMed
Teixeira et al. (2014) 33	Diversity of apolipoprotein E genetic polymorphism significance on cardiovascular risk is determined by the presence of metabolic syndrome among hypertensive patients	Lipids Health Dis	PubMed
Faria et al. (2017) 34	Effects of leptin and leptin receptor SNPs on clinical- and metabolic-related traits in apparent treatment-resistant hypertension	Blood Press	PubMed
Miranda et al. (2013) 35	eNOS polymorphism associated with metabolic syndrome in children and adolescents	Mol Cell Biochem	PubMed
Rodrigues et al. (2017) 36	Decreased comfort food intake and allostatic load in adolescents carrying the A3669G variant of the glucocorticoid receptor gene	Appetite	PubMed
Rocha et al. (2015) 37	Prevalence of the rs1801282 single nucleotide polymorphism of the PPARG gene in patients with metabolic syndrome	Arch Endocrinol Metab	PubMed

 The periodicals’ fields of knowledge revealed an interesting variety of journals from different subject areas, as follows: three on nutrition, two on genetics, two on biology, and eight specifically focused on areas of medicine (hypertension, diabetes, metabolism, and endocrinology). This diversity of fields of knowledge was also observed in the qualifications of the study authors, who were qualified in the following areas: biophysics, nutrition, psychology, psychiatry, medicine, cardiovascular pharmacology, human and molecular genetics, gynecology and obstetrics, toxicogenomics and nutrigenomics, nephrology, endocrinology, cellular and molecular biology, and pharmacology. 

 With relation to the types of study, all publications were the result of cross-sectional, prospective studies with population samples. Eight of these were case-control studies comparing results for a given condition in carriers against “normal” subjects. The samples used in the studies varied in terms of age group: six with adults, five with adolescents, and three with children. 

**Figure 1:**
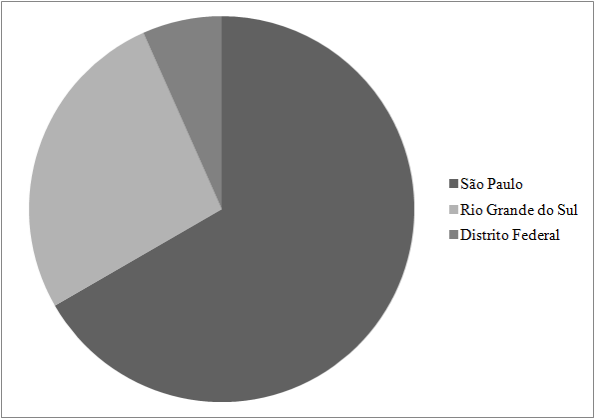
Distribution of articles by states.

 The majority of the studies were conducted in the state of São Paulo (10), followed by Rio Grande do Sul (4) and the Distrito Federal (1). None of the studies were conducted in states in the North or Northeast regions of Brazil ( Figure 1 ). 

## REVIEW

 The foci of identification of the studies selected were investigations of associations between a given genetic polymorphism of a gene involved in the process of a metabolic pathway and risk factors for MS, such as obesity, dyslipidemia, insulin resistance, and hypertension ( Table 2 ). 23 ^-^ 37

**Table 2:** Parameters investigated in the studies selected.

**Authors/data**	**Location of research**	**Sample studied**	**Parameters associated with MS**
Vargas et al. (2013) 23	Rio Grande do Sul	Adults	Lifestyle changes and SNP 48867A> C (Asp358Ala) of the IL6R gene
Belo et al. (2013) 24	São Paulo	Children and adolescents	Polymorphisms of the MMP-2 gene and MS
Brondani et al. (2014) 25	Rio Grande do Sul	Adults	Polymorphism -3826A / G of the UCP1e gene, polymorphism Trp64Arg of the ADRB3 gene and type 2 Diabetes mellitus and MS
Teixeira et al. (2015) 26	São Paulo	Adults	MS with polymorphism 174G/C of the IL-6 gene in hypertensive patients
Almeida et al. (2017) 27	São Paulo	Adolescents	Obesity, insulin resistance and the I/D polymorphism of the ACE gene
Martins et al. (2017) 28	Ribeirão Preto	Adults	SNPs of the GR gene, cytokines, and the hypothalamus-pituitary-adrenal axis
Schuch et al. (2013) 29	São Paulo	Adults	VDR polymorphism, insulin release, insulin resistance, and HDL cholesterol
Gelaleti et al. (2015) 30	São Paulo	Adults and children	Polymorphism Arg972 of the IRS-1 gene, diabetes, and hyperglycemia
Franca et al. (2016) 31	São Paulo	Children and adolescents	Polymorphisms of the APOA5 gene and lipid metabolism
Maintinguer Norde et al. (2017) 32	São Paulo	Adults	SNP of genes IL-6, IL-1β, and IL-10 and plasma fatty acids
Teixeira et al. (2014) 33	São Paulo	Adults	Polymorphism of the ApoE gene and MS in hypertensive patients
Faria et al. (2017) 34	São Paulo	Adults	SNPs rs7799039 and rs1137101 in the LEP and LEPR genes and hypertension
Miranda et al. (2013) 35	São Paulo	Children and adolescents	Polymorphisms of the eNOS gene and MS
Rodrigues et al. (2017) 36	Rio Grande do Sul	Adolescents	SNP A3669G of the GR gene and preferences for palatable foods and metabolic, behavioral, and neural results
Rocha et al. (2015) 37	Brasília	Adults	Anthropometric, biochemical, and hemodynamic variables and SNP rs1801282 of the PPARG gene

 The relationship between differences in the polymorphic frequency of genes involved in obesity and MS was analyzed in five studies with samples of adults and adolescents with different population profiles. One of them found that nutritional control and exercise may be more effective at preventing risks associated with MS in individuals with the A allele for polymorphism 48867A> C (Asp358Ala) IL6R (rs2228145). 23 A study by Belo et al. 24 investigated polymorphisms of the MMP-2 gene and their relationship with metabolic risk factors in obese children and adolescents. The study showed that arterial blood pressure is related to concentrations of MMP-2 in circulation, that the CC genotype of the C polymorphism was more common in both controls and in obese subjects, and that the CT genotype and the T allele for polymorphism C (-735) T are less common in the obese. Brondani et al. 25 evaluated associations between the -3826A/G polymorphism of the UCP1 gene and the Trp64Arg polymorphism of the ADRB3 gene with type 2 Diabetes mellitus and MS characteristics. In this study these polymorphisms were not associated with diabetes, but they may have a combined effect on modulation of excess weight/obesity and on HDL-C levels in Brazilian Caucasian patients with type 2 Diabetes mellitus. Another study stressed the importance of the relationship between the C allele at locus -174 of the IL-6 gene, which was found to be involved in the inflammatory process with occurrence of MS and pathogenesis of visceral obesity. 26 Finally, another study demonstrated that genotypes of the ACE I/D gene can influence regulation of insulin resistance and reduction of cholesterol levels of low density lipoproteins in obese adolescents on long-term multidisciplinary interventions, including medical care, psychological therapy, nutritional programs, and physical exercises. 27

 Three studies with different populations analyzed links between insulin resistance, diabetes mellitus, and MS. One study analyzed the NR3C1 polymorphism, expression of glucocorticoid receptor (GR) isoforms and cytokines, demonstrating that patients with MS exhibited reduced hypothalamus-pituitary-adrenal axis (HPA) sensitivity to glucocorticoid feedback and that dysregulation of this axis could contribute to pathogenesis of MS. 28 An investigation of the relationships between the 2228570 C>T and 1544410 A>G polymorphisms of the VDR gene and MS in adults suggests that they could influence insulin release and insulin resistance, but was unable to determine their influence on components of MS. 29 A study by Gelaleti et al. 30 evaluated presence of the Arg972 polymorphism of the IRS-1 gene in pregnant women with diabetes or mild gestational hyperglycemia and their newborn infants. The results showed that this polymorphism was more prevalent in the newborn infants of women with diabetes and mild gestational hyperglycemia. 

 Associations between genes, lipid metabolism, and MS were investigated in three studies. One of these investigated polymorphism of the APOA5 gene and lipid metabolism, demonstrating that this is a genetic risk factor for MS in children and adolescents. 31 Another investigation noted that the G allele of the IL6 SNP rs1800795 gene was associated with increased probability of MS and that the plasma fatty acid profile interacts with variants of the IL1B and IL10 genes to modulate manifestation of MS. 32 Another study with the ApoE polymorphism, involved in regulation of cholesterol and triglycerides metabolism found an association between the ApoE gene and the syndrome. 33

 De Faria et al. studied relationships between genes and hypertension, 34 but their analyses did not detect direct associations between patients with apparently treatment resistant hypertension and the leptin gene (LEP) or the leptin receptor gene (LEPR). Miranda et al. 35 examined interactions between the eNOS gene and cardiovascular risk and their results suggested that while eNOS haplotypes were not relevant, the CC genotype of the T (786) C polymorphism is associated with MS in obese children and adolescents. 

 A study by Rodrigues et al. 36 tested whether presence of the G variant of SNP A3669G of the GR gene would affect preference for palatable foods and alter metabolic, behavioral, and neural results. Their results showed that the GC allele is associated with reduced sensitivity which, on the cognitive and behavioral levels, results in altered ingestion of foods and response to emotional stress. Additionally, they found that this genetic variant can play an important role in reduction of risk of metabolic and psychiatric diseases. 

 Another study tested for interactions between the PPARG gene and anthropometric, biochemical, and hemodynamic variables in patients with MS. Their results suggested that the rs1801282 polymorphism of this gene is not correlated with predisposition to MS. 37

## DISCUSSION

 Our analysis shows that despite the reasonable number of Brazilian studies investigating the relationship between genes and their polymorphic variants and MS and its risk factors, these studies are published in periodicals and written by authors from a range of areas of knowledge. This diversity is evidence of the importance of increasing knowledge of this subject in several different areas of healthcare. Additionally, the diversity of the subjects who made up the study samples demonstrates the relevance of metabolic abnormalities and cardiovascular diseases at all phases of human development and in groups with distinct populational genetic structures. Nevertheless, it can be concluded that there is a need to increase the number of studies in order to better examine the biological role played by genetic polymorphisms in patients with MS or with its risk factors. 

 With regard to the locations where research is conducted, our searches show that there research is highly concentrated, primarily in the state of São Paulo. This state has the majority of the country’s state universities and, consequently, of the more traditional and most consolidated research centers, which are also those with better infrastructure and finance. It can be observed that Brazil needs more research of this type, considering the importance of the subject, the huge scale of the country, and its highly diverse population structure. 

## FINAL COMMENTS

 In summary, this integrative review has demonstrated that there are insufficient studies investigating the genetic polymorphisms involved in development of MS and its risk factors in samples from the Brazilian population. As a consequence, the results are insufficient to provide an understanding of the complex genetic structure of the county’s racially diverse population. Finally, it is very important that more studies be conducted to attempt to identify the roles and relationships of genetic polymorphisms in manifestations of MS and its risk factors. 
