# Supplementary material for: An integrative review of associations between polymorphic variants and the metabolic syndrome
Source: J Vasc Bras. 2018 Apr-Jun;17(2):141–7. doi: 10.1590/1677-5449.007917 (PMC6205710; doi:10.1590/1677-5449.007917)
Supplement: Translation

# Translation: Associação de variantes polimórficas com síndrome metabólica: uma revisão integrativa

**Authors:** Jamille Silva Oliveira, Rita Narriman Silva de Oliveira Boery

**Affiliations:** 1 Universidade Estadual do Sudoeste da Bahia – UESB, Programa de Pós-graduação em Enfermagem e Saúde – PPGES, Jequié, BA, Brasil.

**Keywords:** polimorfismo genético, síndrome metabólica, hipertensão, obesidade, resistência à insulina, dislipidemia

## Abstract

A patogênese da síndrome metabólica, ou seja, de cada um de seus componentes, é complexa e não totalmente elucidada. Por isso, há grande dificuldade em se estabelecer uma definição de quais fatores clínicos e biológicos seriam os principais determinantes no seu desenvolvimento. Esta revisão tem como objetivo caracterizar a produção científica brasileira que aborda o estudo da síndrome metabólica associada aos fatores genéticos. Foram incluídos 15 estudos, levando em consideração os critérios de inclusão e exclusão. Nossa análise revela uma razoável quantidade de trabalhos brasileiros que investigam a relação de genes e suas variantes polimórficas com a síndrome metabólica e seus fatores de risco. Dessa forma, ressalta-se a necessidade de mais trabalhos que examinem melhor o papel biológico ou a relação dos polimorfismos genéticos em pacientes com síndrome metabólica ou com seus fatores de risco.

## INTRODUÇÃO

 A síndrome metabólica (SM) tem sido utilizada como ferramenta clínica para identificar pacientes com risco metabólico de doenças cardiovasculares 1 . De forma geral, a SM pode ser classificada como um conjunto de fatores fisiopatológicos de risco cardiovascular relacionados metabolicamente com origem em desordens complexas, com influência ambiental e genética 2 ^,^ 3 . A patogênese da SM, ou seja, de cada um de seus componentes, é complexa e não totalmente elucidada. Por isso, há grande dificuldade em se estabelecer uma definição de quais fatores clínicos são os principais determinantes no seu desenvolvimento. Contudo, há um consenso sobre os principais componentes da SM que estão associados ao aumento da morbimortalidade cardiovascular: excesso de peso, aumento da pressão arterial, distúrbios do metabolismo dos glicídios e lipídios 2 ^,^ 4 ^-^ 7 . 

 Por ser um distúrbio multifatorial, o fator genético, assim como o fator ambiental, é importante para o entendimento das interações entre todos os componentes de risco para o desenvolvimento da SM. Nesse contexto, vários lócus gênicos estão envolvidos na expressão dos componentes do metabolismo energético, o que torna complexo determinar a influência das interações gene-ambiente e gene-gene de cada um dos fatores de risco cardiovascular 8 . 

 Pequenas variações na sequência de DNA causadas por mutações pontuais do tipo substituição, denominadas polimorfismos de nucleotídeo único (SNPs), representam a classe de marcadores mais comum no genoma humano, correspondendo a cerca de 90% das diferenças de sequência 9 . Logo, há 3 milhões de diferenças de uma base e cerca de 10.0000 aminoácidos diferentes entre quaisquer duas pessoas 10 . Entretanto, a maior parte das mutações de um nucleotídeo único é rara em uma população, sendo necessário que o polimorfismo apresente uma frequência de pelo menos 1% na população para ser considerado SNP. 

 Um dos principais objetivos de pesquisas com SNP é compreender a genética da variação fenotípica humana e, especialmente, a base genética das doenças humanas complexas 11 ^,^ 12 . Os fatores genéticos podem explicar parte do aumento do risco cardiovascular 13 , o que se justifica pelo envolvimento de diversos genes com o controle de diferentes vias metabólicas. Os mecanismos determinados por alelos gênicos incluem processos inflamatórios, atividade das plaquetas, atividade neuro-hormonais (sistema renina-angiotensina), metabolismo de lipídeos e estresse oxidativo 14 . 

 Estudos genômicos levaram à identificação de muitos marcadores genéticos de risco/proteção cardiovascular em diferentes populações do mundo, destacando-se os genes IL1RA, CD14, PON1, ALOX5AP, loci 9p 21.3, PON1 (Met55Leu e Gln192Arg) 15 ^-^ 18 . Além disso, estudos sugerem que o papel desses fatores de risco cardiovascular pode variar em função do grupo étnico 19 ^-^ 22 . Assim, essas pesquisas indicam que polimorfismos genéticos distribuídos distintamente entre populações de etnias diferentes exercem papel fundamental na presença das diferenças etnicorraciais observadas na SM. 

 Nesse contexto, este estudo tem como objetivo caracterizar a produção científica brasileira que aborda o estudo da associação entre SM e seus fatores genéticos. 

## METODOLOGIA

 Estudo do tipo revisão integrativa de literatura, de caráter descritivo e exploratório, realizado a partir da busca de trabalhos acadêmicos que versam sobre a seguinte temática: pesquisas genéticas atuais sobre a SM no Brasil. 

 A triagem das bases de dados para a pesquisa foi realizada levando-se em consideração que cada base tem sua peculiaridade, área de concentração e enfoque. Dessa forma, foram escolhidas as bases consideradas relevantes ao tema de pesquisa, como SciELO® (*Scientific Electronic Library Online)*, PubMed Central®, desenvolvida e mantida pelo Centro Nacional de Informações Biotecnológicas ( *National Center for Biotechnology Information,* NCBI), e Biblioteca Virtual em Saúde (BVS) Brasil. Além dessas bases de dados, o Google Acadêmico®/Google Scholar® também foi consultado para uma observação mais ampla das publicações e comparação dos resultados com as outras bases. 

 A busca pelos trabalhos foi realizada no mês de junho de 2017 e, uma vez escolhidas as bases, definiu-se a estratégia de busca. Assim, foram determinadas as palavras-chave polimorfismo, síndrome metabólica e Brasil a partir do tema da pesquisa, as quais foram combinadas com operadores lógicos (*booleanos*) a fim de refinar e especificar a pesquisa. Utilizaram-se as palavras-chave em português e inglês de acordo com as exigências da base. 

 A seleção dos trabalhos foi realizada de acordo com os seguintes critérios de inclusão: 1) publicações em revistas ou jornais; 2) publicações que versassem sobre a temática principal (polimorfismo e SM); e 3) publicações nos últimos 5 anos, entre 2013 e 2017. E, para os critérios de exclusão, foram suprimidos trabalhos que após a leitura do título, palavras-chave ou resumo não abordavam o tema e que não se enquadravam nos parâmentros de inserção. 

 Após a seleção dos estudos, segundo os critérios de inclusão e exclusão, as informações dos trabalhos foram inseridas em formulário próprio para registros dos dados coletados. Tal instrumento foi criado em planilha a fim de organizar, agrupar e facilitar a análise dos dados, a interpretação dos resultados e a apresentação da revisão. Este foi constituído por colunas para inserir referência, categoria, objetivo, metodologia, resultados e conclusão do trabalho. 

## RESULTADO

 Os 15 trabalhos incluídos na revisão apresentavam, de forma geral, o propósito de investigar a relação de genes e suas variantes polimórficas com a SM e seus fatores de risco (obesidade, resistência à insulina, hipertensão e alterações no perfil lipídico). Para verificar tal associação, os genes pesquisados foram: eNOS, MMP-2, IL6R, VDR, UCP1, ADRB3, ApoE, IRS-1, PPARG, APOA5, LEP, LEPR, NR3C1, GR, IL1B, IL2, IL4, IL8, IL10, IFNγ, TNFa e ACE. 

 A primeira busca gerou resultados utilizando apenas as palavras-chave combinadas com operadores *booleanos*: “polimorfismo” AND “síndrome metabólica” AND “Brasil” para as quatro bases de dados. A base que apresentou menos resultados foi o SciELO, com apenas um artigo, seguido pelo BVS com 17, PubMed com 38 e Google Acadêmico com 4.430. Para adaptar e refinar nossa análise, a segunda pesquisa foi realizada aplicando o filtro dos últimos 5 anos (2013-2017). Observa-se que o Google Acadêmico® foi a base que apresentou o maior quantitativo de trabalhos, pois possui uma métrica de busca diferente da utilizada pelas outras bases de dados. Assim, essa base foi utilizada para visualizar as possíveis diferenças entre os resultados gerados pelas outras bases, pois cada uma das bases tem sua própria métrica, o que pode gerar resultados distintos, como artigos contidos em uma base e não em outra. 

 Ao aplicar o filtro de disponibilidade e leitura, que consiste em ler o título, resumo e palavras-chave de cada trabalho, foram selecionados 15 artigos ( Tabela 1 ) 23 ^-^ 37 . Os demais foram excluídos da análise, pois não estavam alinhados com o tema da pesquisa e com os parâmetros de inclusão. 

**Tabela 1:** Estudos selecionados após aplicação dos critérios de seleção.

**Autores/data**	**Título**	**Jornal/revista**	**Base de dados**
Vargas et al. (2013) 23	Influence of the 48867A>C (Asp358Ala) IL6R polymorphism on response to a lifestyle modification intervention in individuals with metabolic syndrome	Genet Mol Res	PubMed e BVS
Belo et al. (2013) 24	Effect of metabolic syndrome risk factors and MMP-2 genetic variations on circulating MMP-2 levels in childhood obesity	Mol Biol Rep	PubMed
Brondani et al. (2014) 25	The presence of at least three alleles of the ADRB3 Trp64Arg (C/T) and UCP1-3826A/G polymorphisms is associated with protection to overweight/obesity and with higher high-density lipoprotein cholesterol levels in Caucasian-Brazilian patients with type 2 diabetes	Metab Syndr Relat Disord	PubMed
Teixeira et al. (2015) 26	Association of IL-6 polymorphism -174G/C and metabolic syndrome in hypertensive patients	Biomed Res Int	PubMed
Almeida et al. (2017) 27	Different metabolic responses induced by long-term interdisciplinary therapy in obese adolescents related to ACE I/D polymorphism	J Renin Angiotensin Aldosterone Syst	PubMed
Martins et al. (2017) 28	HPA axis dysregulation, NR3C1 polymorphisms and glucocorticoid receptor isoforms imbalance in metabolic syndrome	Diabetes Metab Res Rev	PubMed
Schuch et al. (2013) 29	Relationship between Vitamin D Receptor gene polymorphisms and the components of metabolic syndrome	Nutr J	PubMed e BVS
Gelaleti et al. (2015) 30	IRS-1 gene polymorphism and DNA damage in pregnant women with diabetes or mild gestational hyperglycemia	Diabetol Metab Syndr	PubMed
Franca et al. (2016) 31	APOA5 polymorphisms associated with lipid metabolism in Brazilian children and adolescents	Genet Mol Res	PubMed
Maintinguer Norde et al. (2017) 32	Influence of IL1B, IL6 and IL10 gene variants and plasma fatty acid interaction on metabolic syndrome risk in a cross-sectional population-based study	Clin Nutr	PubMed
Teixeira et al. (2014) 33	Diversity of apolipoprotein E genetic polymorphism significance on cardiovascular risk is determined by the presence of metabolic syndrome among hypertensive patients	Lipids Health Dis	PubMed
Faria et al. (2017) 34	Effects of leptin and leptin receptor SNPs on clinical- and metabolic-related traits in apparent treatment-resistant hypertension	Blood Press	PubMed
Miranda et al. (2013) 35	eNOS polymorphism associated with metabolic syndrome in children and adolescents	Mol Cell Biochem	PubMed
Rodrigues et al. (2017) 36	Decreased comfort food intake and allostatic load in adolescents carrying the A3669G variant of the glucocorticoid receptor gene	Appetite	PubMed
Rocha et al. (2015) 37	Prevalence of the rs1801282 single nucleotide polymorphism of the PPARG gene in patients with metabolic syndrome	Arch Endocrinol Metab	PubMed

 Quanto à área de conhecimento dos periódicos, identificou-se uma variedade interessante de revistas pertencentes às seguintes áreas: três de nutrição, duas de genética, duas de biologia e oito especificamente da área médica (hipertensão, diabetes, metabolismo, endocrinologia). Essa diversidade na área de conhecimento foi verificada também na formação dos autores dos trabalhos, os quais apresentaram as seguintes formações: biofísica, nutrição, psicologia, psiquiatria, medicina, farmacologia cardiovascular, genética humana e molecular, ginecologia e obstetrícia, toxigenômica e nutrigenômica, nefrologia, endocrinologia, biologia celular e molecular, e farmacologia. 

 Com relação ao tipo de estudo, todos os trabalhos foram resultado de pesquisas transversais, prospectivas e com amostras populacionais. Destes, oito eram trabalhos do tipo caso-controle que comparam seus resultados entre portadores e “normais” para determinada condição. Quanto aos sujeitos que integraram as pesquisas, observa-se que os estudos aplicaram suas análises em indivíduos de faixa etária distinta: seis em adultos, cinco em adolescentes e três em crianças. 

 A maioria dos trabalhos encontrados na busca foi realizado no estado de São Paulo (10), seguido de Rio Grande do Sul (4) e Distrito Federal (1). Nenhum trabalho foi realizado na região Norte e Nordeste ( Figura 1 ). 

**Figura 1:** Distribuição dos artigos por estado.

## REVISÃO

 Identificou-se que os trabalhos selecionados apresentaram como foco a investigação da associação de um determinado polimorfismo genético de um gene envolvido no processo da via metabólica com fatores de risco para SM, como obesidade, dislipidemia, resistência à insulina e hipertensão ( Tabela 2 ) 23 ^-^ 37 . 

**Tabela 2:** Parâmetros pesquisados nos trabalhos selecionados.

**Autores/data**	**Local da pesquisa**	**Amostra estudada**	**Parâmetros associados com SM**
Vargas et al. (2013) 23	Rio Grande do Sul	Adultos	Modificação do estilo de vida e o SNP 48867A> C (Asp358Ala) do gene IL6R
Belo et al. (2013) 24	São Paulo	Crianças e adolescentes	Polimorfismos de gene MMP-2 com a SM
Brondani et al. (2014) 25	Rio Grande do Sul	Adultos	Polimorfismo -3826A / G do gene UCP1e o polimorfismo Trp64Arg do gene ADRB3 com diabetes mellitus tipo 2 e SM
Teixeira et al. (2015) 26	São Paulo	Adultos	SM com o polimorfismo 174G/C do gene IL-6 em hipertensos
Almeida et al. (2017) 27	São Paulo	Adolescentes	Obesidade, resistência à insulina e o polimorfismo I/D do gene ACE
Martins et al. (2017) 28	Ribeirão Preto	Adultos	SNPs do gene GR, citocinas com o eixo hipotálamo-hipófise-adrenal
Schuch et al. (2013) 29	São Paulo	Adultos	Polimorfismo VDR com a secreção de insulina, resistência à insulina e colesterol HDL
Gelaleti et al. (2015) 30	São Paulo	Adultos e crianças	Polimorfismo Arg972 no gene IRS-1 com diabetes e hiperglicemia
Franca et al. (2016) 31	São Paulo	Crianças e adolescentes	Polimorfismos do gene APOA5 no metabolismo lipídico
Maintinguer Norde et al. (2017) 32	São Paulo	Adultos	SNP de genes IL -6, IL-1β e IL-10 e os ácidos graxos plasmáticos
Teixeira et al. (2014) 33	São Paulo	Adultos	Polimorfismo do gene ApoE com a SM em hipertensos
Faria et al. (2017) 34	São Paulo	Adultos	SNPs rs7799039 e rs1137101 nos genes LEP e LEPR com hipertensão
Miranda et al. (2013) 35	São Paulo	Crianças e adolescentes	Polimorfismos no gene eNOS com a SM
Rodrigues et al. (2017) 36	Rio Grande do Sul	Adolescentes	SNP A3669G do gene GR com preferências por alimentos palatáveis, resultados metabólicos, comportamentais e neurais
Rocha et al. (2015) 37	Brasília	Adultos	Variáveis antropométricas, bioquímicas e hemodinâmicas com SNP rs1801282 do gene PPARG

 A relação entre diferenças na frequência polimórfica de genes envolvidos com a obesidade e SM foi analisada em cinco estudos com perfis populacionais distintos, compostos por adultos e adolescentes. Um deles apontou que o controle nutricional e a prática de exercícios poderiam prevenir os riscos associados à SM de forma mais eficiente em indivíduos com o alelo A para o polimorfismo 48867A> C (Asp358Ala) IL6R (rs2228145) 23 . O trabalho de Belo et al. 24 investigou polimorfismos do gene MMP-2 e sua relação com os fatores de risco metabólicos em crianças e adolescentes obesos. Esse estudo demonstrou que a pressão arterial está associada às concentrações de MMP-2 circulantes, que o genótipo CC para o polimorfismo C foi mais comum tantos nos indivíduos controles quanto nos obesos, e que o genótipo CT e o alelo T para o polimorfismo C (-735) T são menos comuns na obesidade. A associação do polimorfismo -3826A / G do gene UCP1 e do polimorfismo Trp64Arg do gene ADRB3 com diabetes melito tipo 2 e características de SM foi avaliada por Brondani et al. 25 . Nessa pesquisa, os polimorfismos não foram associados com o diabetes, mas podem ter um efeito combinado na modulação do excesso de peso/obesidade e dos níveis de HDL-C em pacientes caucasiano-brasileiros com diabetes melito tipo 2. Outro estudo enfatiza a importância da relação do alelo C no lócus -174 do gene IL-6, o qual encontra-se envolvido no processo inflamatório com a ocorrência da SM e a patogênese da obesidade visceral 26 . Por fim, outro estudo demonstra que os genótipos do gene ACE I/D podem influenciar na regulação da resistência à insulina e na redução dos níveis de colesterol de lipoproteínas de baixa densidade em adolescentes obesos submetidos a intervenção multidisciplinar de longo prazo, como acompanhamento médico, terapia psicológica, programas nutricionais e exercícios físicos 27 . 

 Uma ligação genética entre resistência à insulina, diabetes melito e SM foi analisada em três trabalhos de populações diferentes. O trabalho que analisou o polimorfismo NR3C1, expressão de isoformas de receptores de glucocorticóide (GR) e citocinas demonstrou que pacientes com SM apresentaram diminuição da sensibilidade do eixo hipotálamo-hipófise-adrenal (HPA) ao feedback dos glucocorticóides e que a desregulação desse eixo pode contribuir para a patogênese da SM 28 . A investigação da relação dos polimorfismos 2228570 C>T e 1544410 A>G do gene VDR com a SM em adultos sugere que eles podem influenciar a secreção de insulina e a resistência à insulina, porém não consegue determinar sua influência nos componentes da SM 29 . O trabalho de Gelaleti et al. 30 avaliou a presença do polimorfismo Arg972 do gene IRS-1 em mulheres grávidas com diabetes ou hiperglicemia gestacional leve e em seus recém-nascidos. Seus resultados verificaram que o polimorfismo foi mais prevalente em recém-nascidos de mulheres diabéticas e com hiperglicemia gestacional leve. 

 A associação entre genes com o metabolismo de lipídeos e a SM foi investigada em três pesquisas. O trabalho que pesquisou o polimorfismo do gene APOA5 e o metabolismo lipídico demonstrou que este é um fator de risco genético da SM em crianças e adolescentes 31 . Em outra investigação, nota-se que o gene IL6 SNP rs1800795 alelo G está associado a probabilidades aumentadas de SM e que o perfil de ácidos graxos do plasma interage com as variantes do gene IL1B e IL10 para modular a manifestação da SM 32 . Outro estudo com o polimorfismo ApoE, envolvido na regulação do metabolismo de colesterol e triglicerídeos verificou um associação entre o gene ApoE e a síndrome 33 . 

 A relação entre genes e hipertensão foi pesquisada por Faria et al. 34 , que em suas análises não encontraram associação direta entre pacientes hipertensos aparentemente resistentes ao tratamento com os genes da leptina (LEP) e do receptor de leptina (LEPR). Miranda et al. 35 examinaram a interação do gene eNOS com o risco cardiovascular e seus resultados sugerem que, embora os haplótipos de eNOS não sejam relevantes, o genótipo CC para o polimorfismo T (786) C está associado a SM em crianças e adolescentes obesos. 

 A pesquisa de Rodrigues et al. 36 verificou se a presença da variante G do SNP A3669G do gene GR afetaria as preferências por alimentos palatáveis e alteraria os resultados metabólicos, comportamentais e neurais. Seus resultados destacam que o alelo GC está associado com a redução de sensibilidade que, em níveis cognitivos e comportamentais, se traduz em ingestão alterada de alimentos e resposta ao estresse emocional. Além disso, constatou-se que essa variante genética pode desempenhar um papel importante na redução do risco de doenças metabólicas e psiquiátricas. 

 Outra pesquisa verificou a interação do gene PPARG com variáveis antropométricas, bioquímicas e hemodinâmicas em portadores de SM. Seus resultados sugerem que o polimorfismo rs1801282 desse gene não está correlacionado com a predisposição à SM 37 . 

## DISCUSSÃO

 Nossa análise revela que, apesar da razoável quantidade de trabalhos brasileiros que investigam a relação de genes e suas variantes polimórficas com a SM e seus fatores de risco, essas pesquisas demonstram uma variedade de áreas de conhecimentos dos periódicos e dos autores. Essa diversidade reforça e evidencia a importância no aprofundamento do conhecimento desse tema em diversas áreas da saúde. Além disso, a diversificação dos sujeitos que integraram as pesquisas demonstra a relevância das alterações metabólicas e das doenças cardiovasculares em todas as fases do desenvolvimento humano e em grupos com estrutura genética populacional distinta. No entanto, observa-se que é necessário aumentar o número de pesquisas que examinem melhor o papel biológico dos polimorfismos genéticos em pacientes com SM ou com seus fatores de risco. 

 Quanto ao local da pesquisa, nossa busca demonstra o grande fluxo e concentração de pesquisas realizadas principalmente no estado de São Paulo. Esse estado concentra a maioria das universidades estaduais e consequentemente dos centros de pesquisas mais antigos, consolidados, estruturados e melhor financiados nacionalmente. Observa-se que mais pesquisas são imprescindíveis no país devido à relevância da temática, à grande extensão do país e à sua estrutura populacional diversificada. 

## CONSIDERAÇÕES FINAIS

 Em suma, esta revisão integrativa demonstrou que as investigações de polimorfismos genéticos envolvidos no desenvolvimento da SM e seus fatores de risco em amostras populacionais brasileiras são insuficientes. Dessa forma, os resultados são ínfimos para compreender a complexa estrutura genética da população miscigenada do país. Finalmente, ressalta-se a importância de mais trabalhos que busquem identificar o papel ou a relação dos polimorfismos genéticos na manifestação da SM e de seus fatores de risco.
